# You have to let go sometimes: advances in understanding goal disengagement

**DOI:** 10.1007/s11031-022-09980-z

**Published:** 2022-11-08

**Authors:** Cathleen Kappes, Kaspar Schattke

**Affiliations:** 1 Department of Psychology, University of Hildesheim, Universitätsplatz 1, 31141 Hildesheim, Germany; 2 Département de psychologie, Université du Québec à Montréal, C.P. 8888 succursale Centre-ville, H3C 3P8 Montréal, Québec Canada

## Abstract

While research on tenacious goal pursuit and persistence has evoked a myriad of research efforts, research on goal disengagement has rather been neglected and has been focusing mainly on positive consequences of individual differences in goal disengagement capacities. In recent years, however, research on goal disengagement has seen an upsurge in studies, specifically addressing the conceptualization of goal disengagement, the processes involved, and factors facilitating or undermining it. However, many questions remain unanswered or only partly answered providing numerous opportunities for further investigation.

With this special issue of Motivation and Emotion, we aim to stimulate such progress in research on goal disengagement. To this end, this special issue includes empirical studies with cross-sectional, prospective, longitudinal, and experimental designs with a wide range of personal and experimentally induced goals as well as invited commentaries from scholars across different psychological sub disciplines.

In this introductory essay, we provide a brief review of the current state of goal disengagement research. We also provide an overview about the contributions to this special issue with reflections related to the current state of research and areas where further advancement in conceptualization and empirical studies is needed.

The goal concept is central to motivation psychology (e.g., Gollwitzer 2018) as motivation orients, energizes, and selects behavior towards some type of goal or goal state – a notion that many motivation researchers share (e.g., Heckhausen & Heckhausen 2018; McClelland, 1987; Rheinberg & Vollmeyer, 2018; Sheldon & Elliott, 1999). Locke and Latham (1990) even concluded that all motivation would be somehow goal directed. In accord with Austin and Vancouver (1996), we define goals “as internal representations of desired states, where states are broadly construed as outcomes, events, or processes” (p. 338). In addition, goals are characterized as directing our behavior purposefully to attain this desired state (in distinction to wishes and desires; Elliot & Fryer 2008). Given that goals “integrate cognitive, affective, and behavioral processes” (Brandstätter & Hennecke, 2018, p. 453), they play a major role in our everyday life and over the life course (Bühler et al., 2019). Researchers have been approaching the goal concept from many different theoretical angles and with different measures and paradigms. These approaches have inspired a lot of research on how to select goals (e.g., Oettingen et al., 2001; Sheldon, 2014), on how to set and formulate goals (e.g., Locke & Latham 1990), on the relation of means and goals (e.g., Kruglanski et al., 2002), and on how to stick to one’s goals (e.g., Gollwitzer 1999), to name but a few.

The process from setting a goal to achieving it has been famously described in the Rubicon Model of action phases (Heckhausen & Gollwitzer, 1987), which focuses on the two fundamentally different processes (Lewin, 1926) involved in selecting versus pursuing a goal. Specific mindsets characterize each of the different action phases (Gollwitzer, 1990). In the *predecisional phase*, people deliberate the desirability and feasibility of their various wishes before forming an intention of which one to pursue. The accompanying *deliberative mindset* ensures an open minded and objective evaluation of how much people want the potential action outcome and how difficult it is to attain. Once they have made up their mind, they cross the Rubicon into the *preactional phase*, in which they plan their action. The accompanying *implemental mindset* highlights information that facilitates initiating goal-oriented behavior. Whereupon people start realizing their intention in the *actional phase*. The accompanying *action mindset* masks information and thoughts challenging or contradicting the initial decision to pursue the goal and focuses instead solely on sustaining the course of action. In the *postactional phase*, people evaluate the success of their action. The accompanying *evaluative mindset* is directed at assessing information that sheds light on the quality of the achieved action outcomes and the obtained consequences of these outcomes. If people are satisfied, they will deactivate the goal. Otherwise, they will adapt effort or aspiration level and act on the goal again (Achtziger & Gollwitzer, 2018). In their motivational theory of life-span development (MTD), Heckhausen et al., (2010, 2019) extended the model in conceptualizing disengagement from goals, for example, when crossing a deadline characterized by loss of opportunities for (productive) goal pursuit. In the same vein, but independent from the Rubicon model, Brehm and Self (1989; see also Richter et al., 2016; Silvestrini & Gendolla, 2019), in their motivation intensity theory (MIT), considered withdrawal of effort (i.e., resource mobilization) as a function of the interplay between subjective task difficulty – which is related to the expectancy of success – and success importance (with the latter setting the motivation potential, i.e., the maximally justified effort). How does the process of goal disengagement unfold and which factors influence it?

Most of the research has been focusing on processes and factors that ensure successful goal setting, tenacious goal pursuit in the face of difficulties, and goal attainment. For example, Locke and Latham (1990, 2019) established that setting specific and challenging goals leads to increased performance and satisfaction. Moreover, Klug and Maier (2015) have demonstrated a positive relationship between progress towards goal achievement and well-being. Others demonstrated the positive association between persisting and succeeding in several life domains (Credé et al., 2017; Duckworth et al., 2019; Eskreis-Winkler et al., 2014; Tang et al., 2019) and persistence’ relevance for well-being (Disabato et al., 2019). Yet, in some situations persistent goal pursuit might become unfruitful or potentially even maladaptive (e.g., Kalia et al., 2019; Lucas et al., 2015). This would be the case when the pursuit consumes too many resources, a goal is unattainable altogether, or when the goal is not as desired anymore. Therefore, healthy or *functional goal regulation* consists of persistent goal pursuit (goal engagement) *and* flexible goal adjustment (goal disengagement and reengagement) as well as their successful interplay (Brandtstädter & Rothermund, 2002; Baltes, 1997; Haase et al., 2013; Heckhausen et al., 2019; Hommel, 2015). While there is considerable research on the positive consequences of goal disengagement on well-being (Barlow et al., 2020; Heckhausen et al., 2019; Wrosch & Scheier, 2020), research has focused less on antecedents, mechanisms, and processes involved in goal disengagement. Although early research had considered responses to situations of obstacles in goal pursuit (Dembo, 1931; Ovsiankina, 1928; Zeigarnik, 1927), researchers only started in the past 30 years to focus explicitly on goal disengagement (Brandstätter & Bernecker, 2022; Brandtstädter & Renner 1990; Brehm & Self, 1989; Brandtstädter & Rothermund, 2002; Ghassemi & Brandstätter, 2019; Heckhausen, 1997; Richter et al., 2016; Wrosch et al., 2003).

Therefore, the aim of this special issue is to assemble diverse theoretical and methodological approaches to display the status quo of current research and identify future avenues for this rather neglected research topic. Moreover, we have invited several established goal researchers to comment on the empirical articles of this special issue and thereby to extend the scope of the considered theories (Heckhausen & Wrosch, 2022; Oettingen & Gollwitzer, 2022). In this introductory paper, we will first outline different conceptualizations of goal disengagement. Using different theoretical perspectives, we will then discuss the goal disengagement process followed by relevant moderators. We integrate the findings of this special issue’s empirical articles in the following sections. We will finish by pointing open questions out and with proposing avenues for future research.

## Conceptualizations of goal disengagement

During the actional phase of goal pursuit an individual might be confronted with obstacles, might approach or cross a (developmental) deadline, or goal pursuit might take so much time that life circumstances change rendering goal achievement unattractive. The experience of being confronted with an unattainable goal, investing effort in a goal with uncertainty about its expedience, or realizing that a goal might not be worth achieving anymore is a well-known phenomenon. The COVID-19 pandemic has been a particularly stark reminder that sometimes goals cannot be achieved or that their attainability is at least uncertain. Ritchie et al., (2021) reported that during the beginning of the pandemic most of their participants were uncertain or did not believe that they could achieve their goal. Given these uncertainties about whether one’s goal can be achieved, goal disengagement seems to be a suitable response. Indeed, over a quarter of the participants either stopped goal pursuit or voiced uncertainty about pursuing it in the future. At the same time, participants indicated that they still cared about their goals. Thus, did these people actually disengage from their goal and was their goal regulation functional?

Goal disengagement presupposes previous commitment to a goal. According to expectancy-value theory, “commitment […] describes the extent to which personal goals are associated with a strong sense of determination, with the willingness to invest effort, and with impatient striving for goal implementation” (Brunstein, 1993, p. 1062). Based on the assumptions of expectancy-value theory, the extent of the desirability (value) and feasibility (expectancy) of a goal are central to the degree of commitment to a goal (Brandstätter & Hennecke, 2018). Thus, having and committing to a goal “means to direct our behavior purposefully toward something desirable in the future, whose realization we consider positive” (Brandstätter & Hennecke, 2018, p. 453). Importantly, this entails the integration of cognitive, affective, and behavioral processes. Conversely, according to this theoretical perspective, disengagement from a goal then means withdrawing commitment cognitively, affectively, and behaviorally (Brandstätter & Bernecker, 2022; Brandtstädter & Rothermund 2002; Wrosch et al., 2003).

Previous theories on goal disengagement either did not distinguish between the cognitive, affective, and behavioral facet^1^ or emphasized some facet considerably more than the others. For example, theories stemming from an economic point of view (e.g., sunk costs, Arkes & Blumer 1985; entrapment, Brockner et al., 1981; escalation of commitment, Schultze et al., 2012) focused on a lack of disengagement despite decreasing chances of goal achievement or risk of wasting resources with continued goal pursuit. They mainly investigated continued effort in terms of investing resources (e.g., time or money). Within this framework, Henderson et al., (2007) studied increased goal disengagement in terms of disengaging behaviorally from a chosen strategy but without explicitly considering affective and cognitive facets. Moreover, a focus on behavioral measures of disengagement is oftentimes found in experimental studies employing duration or number of working on unsolvable or difficult tasks as indicators of goal commitment (e.g., Kappes & Thomsen 2020; Koppe & Rothermund, 2017; Lench & Levine, 2008; Randenborgh et al., 2010; Richter et al., 2016). Within the MIT (e.g., Richter et al., 2016; Silvestrini & Gendolla, 2019), goal disengagement is assessed as reduced or ceased physiological response or hand grip. Although decrease in or lack of behavioral or physiological effort might signal disengagement, it does not preclude continued affective and cognitive commitment. This might be the case when pausing actual goal pursuit, such as with “frozen”/“shelved” goals (see below). Moreover, in some cases behavioral disengagement cannot be employed as indicator of goal disengagement because further behavioral effort is not possible anymore with definite unattainability of the goal (e.g., due to exceeding a deadline for goal fulfilment) but individuals could still deem goal importance high. Therefore, reduction in behavioral or physiological effort might not be unequivocal in signaling goal disengagement.

In contrast, some research has focused particularly on the dissolution of affective and cognitive commitment employing situation-specific or dispositional measures of cognitive ease in disengaging from a goal (e.g., Ntoumanis et al., 2014; Wrosch et al., 2007). In this vein, Rothermund (2006, p. 226) argued and demonstrated empirically that the reduction of goal importance indicates a disengagement process resulting in the reduction of distress associated with blocked goals (although he maintains that there are other ways, too, such as adjusting previous aspirations or reappraisal of the situation, to reduce distress). In terms of the MIT (Brehm & Self, 1989; Richter et al., 2016), lower goal importance then decreases the level of maximally justified effort (i.e., lower potential motivation), possibly reducing the intensity of exerted effort to zero.

Overall, previous research has rarely considered the various facets of goal disengagement and their interrelationship within one study and across time. The different facets of goal disengagement might be differentially associated with measures of functionality, such as well-being. Moreover, antecedents of goal disengagement might affect these facets differently. Finally, the facets might interact with each other, for instance, behavioral disengagement might allow physical distance from the goal and set affective and cognitive disengagement into motion, whereas a reduction in affective commitment might influence behavioral disengagement.

Hubley and Scholer (2022, in this volume) address the issue of asynchronous disengagement regarding so-called frozen goals (Davydenko et al., 2019), that is, goals for which effort is withdrawn but there is continued affective and cognitive commitment. As pointed out, the COVID-19 pandemic disrupted goal pursuit (e.g., due to restrictions in mobility or financial issues related to job loss). Hubley and Scholer (2022) found that ill-being (greater stress, depression, and anxiety) was positively associated with the percentage of frozen goals as well as ruminating about a frozen goal. One could interpret these findings in the sense that behaviorally disengaging from goals without disengaging affectively and cognitively is detrimental to well-being. However, several contextual variables need to be considered that could be relevant for coping with obstacles in goal pursuit and further progress in goal disengagement. For example, it may be relevant whether people perceive obstacles in goal pursuit as permanent or temporary. In the case of temporary behavioral goal disengagement, perceiving the interruption as self-selected and having control about the further pursuit might play an important role.

Mayer and Freund (2022, in this volume) focused on the effect of withdrawing behavioral effort on goal desirability and experienced regret as a function of permanence of effort withdrawal. They experimentally varied the permanence of withdrawing behavioral effort from one of two conflicting goals (scarce resource time) by either “shelving”^2^ the goal, that is, temporarily withdrawing effort with the prospect of continuing later, or disengaging from the goal for good. Both goal shelving and disengagement resulted in a decrease of experienced goal conflict. However, shelving was related to less anticipated as well as experienced regret about withdrawing effort from the goal. The subjective value of the shelved/disengaged goal decreased in both groups but to a larger extent in the group that was permanently prevented from goal pursuit. Interestingly, participants in the “shelving” group consisted of two subgroups, which became visible when given the opportunity to either continue with the prioritized goal or work on the previously shelved group. Participants who reengaged with the shelved goal had only a slightly decreased goal value of the shelved goal after the shelving decision. In contrast, participants who disengaged for good reported lower subjective goal value of the shelved goal after the prioritization decision (comparable to the score of participants in the disengagement condition) and continued goal devaluation later. The subgroups also differed in their asymmetry of the conflicting goals’ values at the beginning of the study with those reengaging in the shelved goal reporting a lower asymmetry.

Even though these findings are not directly comparable to Hubley and Scholer’s (2022), they emphasize the importance of conceptually and empirically distinguishing between different facets of goal disengagement. Namely because disengagement might progress differently for these facets depending on various factors: While participants all withdrew effort from one goal, the pattern in the affective commitment was different depending on the permanence of goal pursuit obstruction. However, even if obstruction was only temporary, some participants reported lower goal value of the shelved goal nonetheless and did not reengage with it. Thus, shelving a goal might alleviate immediate distress of goal conflict and allow either reengagement with the goal at a later point in time or be the starting point for affective disengagement in the process of goal disengagement.

## The process of goal disengagement

Interestingly, the term “goal disengagement” is oftentimes used interchangeably to refer to the process of dissolving commitment as well as to its result, that is, experiencing no commitment to the goal anymore. Here, we want to focus on the process of goal disengagement. As Ghassemi and Brandstätter (2019) as well as Heckhausen et al., (2019) pointed out, research on how the process of goal disengagement unfolds is scarce. As highlighted in the previous section, goal disengagement might follow an asynchronous time course for different facets. Moreover, it is conceivable that in some cases it can be an abrupt shift in commitment (Heckhausen et al., 2010, 2019), while it might be a longer lasting and potentially wavering process in other cases (Brandstätter et al., 2013; Brandtstädter & Rothermund, 2002; Klinger, 1975). Based on the resource conservation principle (Gibson, 1900, as cited in Silvestrini & Gendolla 2019), motivation intensity theory (Brehm & Self, 1989) as well as emotional intensity theory (Brehm, 1999) posit that “*effort rises with subjective task difficulty as long as success is possible and the necessary effort is justified* [italics used by authors]” (Silvestrini & Gendolla, 2019, p. 118). In the case of known task difficulty, the goal’s importance (serving as justification) only defines the level of maximally justified effort (i.e., potential motivation/emotion intensity) without directly influencing actual effort intensity. By contrast, in the case of unspecified difficulty, exerted effort is proportional to the level of goal importance. Effort is predicted to sharply drop if the goal is either perceived to be unattainable or goal importance does not justify exerting the necessary (further) effort. Although many studies found support for these assumptions, there are also conflicting findings (Richter et al., 2016). Still, both theories provide clear predictions about the investment of effort in situations with varying degrees of knowledge about and differences in task difficulty and goal importance which should inform further research. Yet, the studies did not examine the process of goal disengagement over time, that is, how effort and goal importance change in response to an (unexpected) increase in task difficulty (in the case of previously known difficulty) or eventually perceiving the task to be too difficult or resource-consuming (in the case of unknown difficulty) and not being worth the effort anymore.

Klinger (1975) was one of the first to explicitly describe the disengagement process. He proposed an incentive-disengagement-cycle characterized by four phases of an “orderly process of ending commitment to an incentive” (p. 8): *invigoration*, *aggression*, *depression*, and *recovery*. In response to difficulties in goal pursuit (i.e., decrease in attainability) people first increase effort to achieve the goal (*invigoration*) and simultaneously increase the blocked goal’s desirability. Upon realization of continued difficulties despite increased effort, anger and frustration unfold (*aggression*), which transition into sadness and resignation (*depression*) if (perceived) goal attainability continues to be out of reach (i.e., low expectancy of control, Brandtstädter & Rothermund 2002). The cycle ends with the *recovery* phase, in which people commit to new goals.

### Action crisis

An approach focusing on the transition from goal engagement to disengagement is called *action crisis* (Brandstätter et al., 2013; Herrmann & Brandstätter, 2015). An action crisis is characterized by a decisional conflict of whether to maintain a goal or disengage from it. Action crises usually develop after repeatedly encountering obstacles during goal pursuit, which people experience as increasing doubts about the feasibility of their goal. They ponder whether to continue pursuing the goal or to abandon it, which is often accompanied by decreased psychological well-being and health (Herrmann et al., 2019; Holding et al., 2017). Theoretically, action crises are embedded in the Rubicon Model (see above) in the sense that people experiencing an action crisis oscillate between the *predecisional* and the *preactional* phases, thus mingling the implemental with the deliberative mindset (Brandstätter & Schüler, 2013). In other words, they go back and forth between weighing the attainability and desirability and focusing on how to implement the goal (Brandstätter & Schüler, 2013). Over time, experiencing an action crisis is oftentimes associated with a decrease in goal desirability and attainability, while particularly the decrease in goal desirability is associated with an increase in well-being (Ghassemi et al., 2017). Although this approach provides a vivid description of the disengagement process, it leaves some questions unanswered: the time course of disengagement regarding the behavioral, cognitive, and affective facet might be asynchronous. Moreover, it is unclear how the transition from engagement to disengagement essentially unfolds.

### The role of emotions in the process of goal disengagement

Research on the experience of an action crisis has provided insight into cognitive processes associated with the transition from the action phase to goal disengagement. Complementary, Heckhausen et al., (2019) emphasize the pivotal role emotions play in the regulation of action. Emotions can constitute (*a*) an incentive for goal pursuit (*motivational pull*), (*b*) they inform how to deal with an unsatisfactory situation concerning goal achievement (*motivational push*), and (*c*) they balance the effective pursuit of longer-term goals (*motivational resource*).

Regarding emotions as an incentive, Silvestrini and Gendolla (2019) argued “that positive incentive can justify and thus outweigh the aversive aspect of effort” (p. 120). Moreover, Klinger (1975) argued that cues to the positive incentive value of the unattainable goal in the form of reminders could “generally retard the progress of disengagement” (p. 14). Concurringly, Heckhausen (1997) considered imagining positive incentives of goal achievement as one of the selective secondary control strategies to support goal achievement in the face of difficulties. Accordingly, the recurring confrontation with the lost incentive might impede goal disengagement. Overall, this aspect has received less attention in systematic research on goal disengagement.

Instead, recent research has particularly focused on the second and third aspect of emotions in informing the process of goal pursuit and goal disengagement and promoting either process (Gendolla, 2012; Moors et al., 2017; Kunzmann et al., 2014). For example, Ghassemi et al., (2021) showed in a recent experience sampling study that individuals in an intense action crisis experienced doubts particularly in response to setbacks. At the same time, the experience of positive goal-related events was associated with fewer doubts, and this was related to a less intense action crisis in an exploratory analysis. However, still experiencing scattered positive events concerning the goal (besides increasingly negative experiences) might be what holds individuals in an action crisis loop protracting the disengagement from the goal.

In monitoring goal progress, individuals evaluate this progress against criteria as a signal for how to proceed (see below). The affective experiences resulting from evaluations of goal progress might not just be an epiphenomenon but have been demonstrated to be functional in terms of disengagement as well (Carver & Scheier, 1990; Gendolla, 2000; Moors et al., 2017; Klinger, 1975; Kunzmann et al., 2014; Silvestrini & Gendolla, 2019). While negative affect might result from detecting progress below the expected criterion, the concrete resulting affective response differs between individuals and situations. This has been demonstrated to having differential effects for goal regulation, which promotes overall effective goal pursuit in providing resources or preventing resource exploitation. An appraisal of control over overcoming the obstacle and achieving the goal is related to experiences of anger and irritation. In contrast, appraising the situation as indicating low control and lack of resources to overcome obstacles is related to experiences of sadness and resignation. Appraising the situation in a specific way gives rise to further processes; either supporting goal pursuit (e.g., anger prompting more effort and reactant increase of goal desirability) or disengagement (e.g., sadness decreasing energy for continued effort and loss of interest resulting in goal devaluation). Moreover, Gendolla (2012) demonstrated in his implicit-affect-primes-effort-model building on motivation intensity theory (Brehm & Self, 1989) that for the application of emotion knowledge, experiencing feelings is not necessary but can also be activated using emotion primes resulting in the same effects as described above.

Additionally, emotion regulation might be warranted in effective goal regulation to benefit from the functions of emotions (Frijda, 2009). While anger may be conducive to achieving a goal despite obstacles (e.g., Kim et al., 2015) or sadness may support goal disengagement (Barlow et al., 2022), experiencing rage or depression might rather be harmful. Thus, better emotion regulation capabilities should benefit successful goal pursuit but also better goal disengagement. In this regard, Marion-Jetten and colleagues (2022) have shown that people with difficulties in emotion regulation were more likely to develop an action crisis. More precisely, mindfulness seems to be beneficial for adopting adaptive emotion regulation strategies during an action crisis (Marion-Jetten et al., 2021). At the same time, people high in mindfulness were sticking to their goals for longer (Marion-Jetten et al., 2022, Study 3). Thus, it seems that regulating one’s emotions is important when difficulties in the goal pursuit arise. However, in order to become aware of these difficulties, some sort of monitoring of goal progress must come into play.

### Monitoring of goal progress

Harkin et al., (2016) have shown in a meta-analysis that monitoring goal progress promotes goal attainment. However, the findings of Ghassemi et al., (2021) demonstrate that it also depends on whether progress can be detected. As criteria for evaluation, individuals used an intraindividual comparison (i.e., progress rate compared to one’s own usual rate) as well as an interindividual comparison (i.e., progress rate compared to others’ progress rate). In both cases, a smaller progress rate was related to more doubts as part of an action crisis. Concurringly, research on conflict monitoring (i.e., monitoring “the co-occurrence of competing representations in a given situation”, Silvestrini & Gendolla 2019) provides evidence that conflict is experienced as aversive and associated with respective adjustment processes.

Kreibich et al., (2022, in this volume) present findings that might mitigate the effect of detecting slower than usual or smaller relative progress on action crisis. They argue that people high in self-awareness are more likely to monitor goal progress. In their study, they demonstrated that self-awareness supports dealing with difficulties in goal pursuit via its positive link with problem-solving orientation resulting in lower action crisis. This finding corroborates research on the effect of self-awareness (either explicitly or implicitly induced state of self-awareness or dispositional) within the motivation intensity theory research (for an overview, see Silvia 2015). Given a difficult task or unfixed task difficulty, self-aware participants were willing to invest more effort than those who were not self-aware. Interestingly, Kreibich et al., (2022) also found a direct positive relationship between self-awareness and action crisis when controlling for problem-solving. The authors discuss this finding with reference to the relationship between self-awareness and rumination (e.g., Silvia & Phillips 2011) and the link between rumination and action crisis (Brandstätter et al., 2013; see also Hubley & Scholar, 2022). This indicates that the effect of self-awareness might vary depending on the applicability or availability of problem-solving strategies in contrast to strategies that increase action crisis or support goal disengagement when goal pursuit is futile.

### Intentionality of goal disengagement processes

Although monitoring goal progress is per se a conscious process, it can also be unconscious (e.g., Aarts & Custers 2012). For example, people can have acquired triggers that elicit automatic behavioral responses, of which people are often not aware (Aarts & Custers, 2012). The latter points to the question whether an action crisis must necessarily be experienced, a conscious decision to disengage must be taken, or whether goal commitment can dissolve unconsciously: In which sense is goal disengagement an intentional or unintentional process?

While Heckhausen et al., (2010, 2019) maintain that stopping goal pursuit and dissolving commitment can be intentional, Brandtstädter and Rothermund (2002) state that “we cannot disengage from blocked goals through a deliberate decision, nor can we adopt beliefs or valuations that would support such disengagement through an intentional act” (p. 123). Given this perspective, the non-intentional response in valuation processes would then also be true for the *invigoration* phase (or “assimilative” coping in Brandtstädter and Rothermund’s terminology) as we cannot intentionally and reactantly increase the value of a blocked goal either. Notwithstanding, as Brandtstädter and Rothermund point out, it is possible to employ self-management strategies that influence the probability of goal disengagement (see below). Importantly, Brandtstädter and Rothermund (2002) ascertain that “if the person resolves to drop a blocked goal or plan, however, this decision already involves a change in preferences” (p. 123).

Two studies in this special issue used experimental paradigms, in which participants were prevented from maintaining behavioral effort towards an experimentally induced goal. These studies allow us to investigate causal effects of behavioral disengagement without the individual’s explicit decision to disengage. For example, Rühs et al., (2022, in this volume) first induced and then experimentally blocked a social approach goal: participants were prevented from investing further effort into goal pursuit (i.e., no own decision to behaviorally withdraw from the goal), which resulted in a lower perceived goal attainability. As an outcome, negative affect increased, and goal desirability decreased. Moreover, a progressing decrease in goal desirability was associated with a recovery from negative affect at a later point in the procedure (but not with an increase in positive affect or need fulfilment).

As described in the previous section, Mayer and Freund (2022) presented findings from a goal prioritization paradigm showing that being prevented from further goal pursuit was associated with subsequent goal devaluation. This effect was larger for permanent withdrawal from goal pursuit than for temporary withdrawal (“shelved goal”). Ensuing, a greater devaluation of the shelved goal in the group with temporary behavioral disengagement was related to the decision to permanently disengage from this goal. These findings suggest that an explicit decision to disengage is not necessary to set goal disengagement in motion.

While the initiation to disengage might be unintentional, the perceived reasons for disengaging might still have an effect. Therefore, Holding et al., (2022, in this volume) investigated in two prospective studies how the perceived reasons for the decision to disengage were related to perceived progress in disengagement. Artfully, these authors conceptualized disengaging from a goal as its own goal. Consequently, one can also experience an action crisis for the disengagement goal, which they call an “inaction crisis”, that is, questioning the goal to disengage. Their findings point out that resolving to drop a goal does not necessarily mean that the goal disengagement is complete. For example, if participants perceived the decision to disengage from their goal as being out of shame for not having disengaged earlier, felt pressure to disengage, or for other external reasons (controlled goal disengagement), they reported higher inaction crisis and lower progress in goal disengagement. In contrast, perceiving the to-be-disengaged goal as not reflecting core values or identity anymore (autonomous goal disengagement) was associated with a lower inaction crisis and higher perceived progress in goal disengagement. Interestingly, retrospectively reported previous goal importance was irrelevant for the perceived reasons to disengage. Future work is necessary to investigate factors that support perceiving autonomous reasons for goal disengagement as these already entail goal adjustment processes. In addition, future research should investigate the extent to which the perceived reasons for disengaging from a goal differ from the actual experience of autonomy, external control, and motivation strength during the disengagement process (“motivational mindsets”, Meyer et al., 2022).

Overall, these findings suggest that a decision to disengage is neither necessary nor sufficient to disengage. On the one hand, deciding to disengage can be countered by perceiving controlled reasons for disengaging (Holding et al., 2022). Therefore, the decision to disengage needs to be supplemented with corresponding behavior and cognitions to support affective and cognitive disengagement (Brandtstädter & Rothermund, 2002). On the other hand, being prevented from continued goal pursuit can result in goal devaluation, that is, resolving affective commitment, nonetheless (Mayer & Freund, 2022; Rühs et al., 2022).

Moreover, it is possible to just realize that a goal is not important anymore without having explicitly decided to disengage beforehand. Most of the research on goal disengagement focuses on situations where goal desirability is high, but goal pursuit is (permanently) obstructed. Dissolving affective and cognitive commitment is mostly warranted in this case. In contrast, changes in value might occur due to changed life circumstances or perspective on life (cf. Holding et al., 2022). Still, once the decreased goal desirability is realized, it might be difficult to behaviorally disengage from these goals or situations involved in previous goal pursuit with associated psychological, physical, and societal costs. For instance, individuals might continue working for a previously highly valued company or remain in a relationship due to financial dependencies despite having internally resigned.

## Moderators of goal disengagement processes

Although goal disengagement may provide a route to salvage negative consequences of goal unattainability and frees resources for alternative goal pursuit, letting go is sometimes difficult and individuals also differ in their capacity to disengage from goals (Wrosch et al., 2003). Given that perceptions of goal attainability and goal desirability are at the core of goal engagement and disengagement processes, considering factors that influence their evaluation is relevant to further understanding of goal disengagement. Goal desirability as well as attainability are determined by situational (e.g., incentive structure, obstacles) as well as personal factors (e.g., implicit motives, personal values, ability; for an overview see Brandstätter & Hennecke, 2019; Richter et al., 2016). However, these factors have been mainly investigated with respect to studying successful goal pursuit and persistence. Is their opposite manifestation predictive of adaptive and successful goal disengagement or do the same factors have different effects depending on circumstances? Moreover, are there other factors supportive for goal disengagement than for persistent goal pursuit? In the following, we will primarily consider factors which have been addressed in the empirical articles of this special issue to answer these questions. Other factors are, for instance, mental contrasting (Oettingen, 2012; see also Oettingen & Gollwitzer 2022, in this volume), fatigue (Wright, 2014), perceived ability (Wright & Dill, 1993), self-justifcation (Staw, 1997), or publicity of goal commitment (e.g., Kiesler et al., 1974), and impression management motives (Staw,1997).

### Self-control

Dispositional optimism and self-control have been frequently studied regarding their positive relationship with persistence (meta-analysis: de Ridder et al., 2012; review: Rasmussen et al., 2006). High manifestations of optimism and self-control could thus be detrimental to goal disengagement. However, Aspinwall and Richter (1999) demonstrated in a study, employing unsolvable anagrams, that these factors’ effects depended on specific situations. Most participants worked on unsolvable trials until the end of the provided time in the absence of an alternative. However, when participants had the option to choose alternative tasks, those high in optimism or self-mastery beliefs, behaviorally disengaged markedly quicker from the unsolvable trials. Moreover, if the alternative and solvable task was presented as a task testing a different ability, optimists outperformed participants with lower optimism. The findings of Barber et al., (2012) strike a similar note. Here, the effect of dispositional self-control was dependent on dispositional self-awareness and actual task progress. Given high self-awareness, greater self-control was linked to higher probabilities of persistence, whereas it was related to lower persistence when task progress was low. The findings of Kreibich et al., (2022) of an indirect negative effect of self-awareness on action crisis via problem-solving might refer to situations in which difficulties in goal pursuit could be solved with problem-oriented strategies. In contrast, the direct positive effect between self-awareness and action crisis might indicate situations where disengagement might be warranted.

In their contribution to this special issue, Bieleke et al., (2022, in this volume) have studied individual differences in general self-control in two cross-sectional correlational studies. The findings are rather inconsistent and depended on the inclusion or exclusion of boredom-related variables. Under circumstances of goal adjustment in everyday life, higher general self-control showed a significantly positive association with goal disengagement, which disappeared after including boredom variables. In contrast, during the COVID-19 pandemic, general self-control showed a negative relationship with goal disengagement only if boredom variables were included in the analysis. These findings point to the importance of further, potentially moderating factors on the effect of self-control on goal disengagement.

### Implementation intentions

Bieleke et al., (2022) also studied if-then planning (i.e., implementation intentions; Gollwitzer 1999) as a more concrete self-control strategy. Under everyday life circumstances, if-then planning was associated with lower goal disengagement, whereas there was no significant relationship during the COVID-19 pandemic. The authors interpret this differential effect of if-then planning as being “in line with the “flexible tenacity” commonly associated with the automating effects of if-then planning on behavior” (p. 11). Indeed, Legrand et al., (2017) demonstrated that – despite its facilitation of goal attainment (Gollwitzer & Oettingen, 2019) – if-then planning supports dissolution of commitment concerning the behavioral, cognitive, and affective facet when encountering excessive costs in goal pursuit. In the same vein, Henderson et al., (2007) showed that if-then planning could also be used to effectively disengage from an unsuccessful strategy if expanded to include criteria for disengagement when encountering difficulties in goal pursuit. Importantly, *reflection implementation intention*, that is, thinking about the suitability of the chosen strategy when encountering negative feedback, allowed being sensitive to information about the course of goal progress in deciding whether to switch strategies or stick with the strategy. In contrast, *action implementation intention* for disengagement, that is, disengaging after encountering a specific stimulus, also increased disengagement compared to a simple “Do your best”-intention when encountering the set criterion of negative feedback. However, with this intention, participants were no longer sensitive to cues of improved goal progress. These results illustrate that the effect of if-then planning depends on the concrete implementation intention, and at least some individuals who use such strategies frequently may also be able to adapt their implementation strategies to circumstances such as the COVID-19 pandemic. Importantly, as with self-control, implementation intentions as a strategy, shown to be supportive of persistence (Gollwitzer & Oettingen, 2019), can also facilitate goal disengagement given specific circumstances.

### Goal motivation

Another factor that has been studied regarding goal setting and persistence, but also concerning its effect on goal disengagement, is autonomous versus controlled goal motivation as elements of self-determination theory (Ryan & Deci, 2019; Sheldon & Elliot, 1999). Autonomous motivation refers to goals pursued because they are interesting or joyful or represent the individual’s values. In contrast, controlled motivation refers to goals pursued to comply with externally controlled rewards, to avoid sanctioning or felt pressure, or out of obligation. Autonomous goal motivation has been shown to predict greater persistence in various domains (Howard et al., 2021; Vansteenkiste et al., 2020). In contrast, Ntoumanis et al., (2014) studied the effect of autonomous versus controlled goal motivation on goal disengagement in a laboratory study with an unattainable goal. They found that autonomous goal motivation made it harder to disengage mentally (e.g., stop thinking about the goal) despite disengaging behaviorally. The loss of goals whose pursuit is experienced as joyful or individuals identify with and which fulfil the individuals’ needs is more difficult to overcome. However, Ntoumanis and colleagues also found that if participants perceived the goal’s unattainability earlier in goal pursuit, they were more likely to invest into alternative goal pursuit. Accordingly, Ntoumanis and colleagues emphasized the relevance of processes that support the early detection of unattainability, resource conflicts, or exploitation of resources and processes to act upon this detection. They have proposed mental contrasting combined with implementation intentions in their Tripartite Model of Goal Striving (Ntoumanis & Sekidides, 2018).

Moreover, Holding et al., (2022) demonstrated the importance of perceived autonomy in the ending of goal pursuit for progress in goal disengagement (see also Holding et al., 2020). Their findings could be integrated with research concerning proactive goal disengagement, that is, planning the stop of goal pursuit in the future, which has only recently received more attention (concerning retirement: Zacher et al., 2021; see also Aspinwall 2005, on proactive coping). Individuals anticipate future states, available resources, contextual constraints (such as biological or societal deadlines), and potential changes in values and prepare for letting go. These processes may assist the actual behavioral disengagement and dissolution of affective and cognitive commitment once the time has come. This anticipation and preparation might facilitate the perception of autonomous goal disengagement as described by Holding et al., (2022) once it actually unfolds.

### Mindfulness

Another potentially helpful factor in evaluating goal attainability and adjusting goal importance, might be mindfulness. As Ryan et al., (2021) maintain, “being mindful of the present, free of defenses and judgments, allows information to flow and for what is pertinent to become clearer and more salient” (p. 302). On the one hand, this makes selection of autonomous goals more likely and their pursuit more efficient resulting in less action crisis (Marion-Jetten et al., 2022) and higher goal progress and attainment (Donald et al., 2020; Kappes et al., 2022; Smyth et al., 2020). For example, Marion-Jetten and colleagues (2022) showed that students’ and employees’ dispositional mindfulness related positively to autonomous goals, which, in turn, translated into less action crises over time. In addition to facilitating self-concordant goal selection in the predecisional phase of the Rubicon Model, dispositional mindfulness also facilitates coping with obstacles during the goal pursuit in the implementation and action phases. Accordingly, emotion regulation also mediated the relation between dispositional mindfulness and action crises over time in the aforementioned study. Moreover, in an experimental study, a brief mindfulness manipulation led to more adaptive emotion regulation strategies for an induced action crisis for a self-set personal goal. These results persisted even when controlling for goal motivation (Marion-Jetten et al., 2021).

On the other hand, mindfulness might support unbiased monitoring and evaluation of goal attainability or excessively costly goal pursuit and facilitate goal disengagement even for autonomously pursued goals. For example, studies on open-monitoring meditation (i.e., being non-reactive and non-judgmental to possible upcoming thoughts and emotions) revealed a broader attentional scope (Slagter et al., 2007), facilitation of coping with unexpected events (Valentine & Sweet, 1999), and promotion of divergent thinking and cognitive flexibility (Colzato et al., 2012). These factors might facilitate goal disengagement (Brandtstädter & Rothermund, 2002; Hommel, 2015).

### Availability of alternative goals and goal reengagement

The ease of the disengagement process might also depend on the availability of alternative incentives or the substitutability of the goal (Klinger, 1975; Shah & Kruglanski, 2002). For example, Aspinwall and Richter (1999) provided empirical evidence that individuals high in optimism and self-mastery behaviorally disengaged faster from unsolvable anagrams if an alternative task was available. The substitutability of goals is also dependent on the goal structure with subgoals being easier to disengage from than higher level goals, if they are not strongly linked to the focal goal (Kruglanski, 1996).

Related to the availability of alternative goals is the process of reengaging in new goals. Brandstädter and Rothermund (2002) conceptualize goal disengagement and reengagement as parts of the accommodative mode – both alleviating distress due to unsuccessful goal pursuit. In contrast, Wrosch et al., (2007) distinguish between the two processes. A recent meta-analysis provided tentative evidence for their differential effects. While goal disengagement was predictive of lower negative affect and higher quality of life, reengagement was predictive of lower negative and higher positive affect as well as higher quality of life and generally showed stronger associations.

Bieleke et al., (2022) examined predictors of goal reengagement. They found that if-then planning was linked to better reengagement. Moreover, boredom proneness was related to poorer reengagement while boredom avoidance and escape tendencies were associated with better reengagement. In contrast, both boredom factors were unrelated to goal disengagement lending further support for the relative independence of the two processes.

Additionally, Timar-Anton et al., (2022, in this volume) investigated the consequences of disengagement for reengagement. In particular, they have studied the effects of experiencing an action crisis prior to disengaging from this goal on commitment to a new goal as well as its goal motivation. Moreover, they compared effects of disengaging from a goal with continued goal pursuit, and goal attainment on a reengaged goal. While commitment for a goal prior to disengagement was decreased, commitment and controlled motivation to a new goal was then increased. Moreover, the intensity of an action crisis was associated with increases in commitment and autonomous goal motivation.

Timar-Anton et al., (2022) point to the relevance of considering multiple goals and their interdependence (sequentially). In general, goal regulation processes take place in a larger context of multiple goal pursuit (Kruglanski, 1996). The number and quality of alternatives might, on the one hand, support goal disengagement and reengagement processes, but, on the other, they might also create the necessity to disengage from goals in the first place due to conflicting goals or limited resources (Gray et al., 2017; Mayer et al.).

### The role of social relationships

Moreover, as Fitzsimons and Finkel (2018) point out, goal pursuit rarely transpires in isolation but is deeply embedded within social relationships. Research on the role of social relationships in goal regulation has predominantly focused on its role for successful goal pursuit. However, although perceiving the availability of social support has been demonstrated to be conducive to goal progress (e.g., Lee & Ybarra 2017; Vowels & Carnelley, 2022), receiving support can also have negative consequences on affect and goal attainment, for instance, when received unrequested (Kappes & Shrout, 2011).

Moreover, the role of social partners influencing each other in how to cope with unattainable goals, has rarely been considered explicitly. Thomsen et al., (2017; Kappes & Thomsen, 2020) have conducted experimental studies demonstrating that romantic partners not only serve as role models in persistent behavior but also in disengagement from a futile task. In their contribution to this special issue, Light and Chodos (2022, in this volume) have investigated the effect of social support for goal pursuit when experiencing an action crisis in an experimental and a correlational study. Their results demonstrated that being in an action crisis was associated with more negative appraisals of social support, but it was unrelated to positive appraisals. Moreover, when receiving goal support during action crises, experiencing an action crisis was linked to more negative emotions and depressive symptoms. These findings provide further evidence for the two-sided impact of social support on goal regulation. Importantly, however, social support in these studies was measured as directed at supporting goal pursuit. It would be interesting whether social support directed at disengaging from a goal or simply providing support in enduring an action crisis with its uncertainties could decrease negative and increase positive emotions.

## Summary and future directions

In summary, goal disengagement is a process that unfolds in an asynchronous way concerning three different facets: cognitive, affective, and behavioral. So far, researchers have usually conceptualized goal disengagement either for the behavioral (ceasing goal-related actions) *or* for the cognitive and affective facet (withdrawing commitment). Future research needs to consider all three facets together as well as their respective change over time, as it is likely that one can only properly complete the goal disengagement process when one has successfully disengaged concerning all three facets. Of course, this raises the question of how we can determine that the process is indeed complete, which has received little attention so far.

Important determinants of goal pursuit and disengagement are the desirability and the attainability of the goal in question, which are also central elements in the predecisional phase of the Rubicon Model. However, while the Rubicon Model describes the process of goal pursuit from goal setting to the evaluation of goal success, the model is not particularly specific about how the process of disengaging from unsuccessful goal pursuits unfolds. The same is true for Motivation Intensity Theory (e.g., Brehm & Self 1989; Gendolla et al., 2019; Richter et al., 2016). This theory provides clear-cut predictions and empirical support for the investment of effort (i.e., goal engagement). The most important determinants are goal importance and task difficulty (distinguishing between task difficulty that is known and fixed, unknown or unclear, and self-chosen task difficulty (unfixed)). The theory assumes that people drop a goal that surpasses a threshold of manageable difficulty or if the goal is not important anymore. However, to our knowledge, this *change* in difficulty or goal importance and its consequences has not been studied empirically yet in terms of the MIT. Nevertheless, it would be very interesting to apply the basic paradigm in a slightly modified form to study goal disengagement processes.

In contrast, conceptualizations that explicitly focus on goal disengagement processes are Klinger’s (1975) incentive-disengagement-cycle as well as the experience of an action crisis, which often (but not always) leads to goal disengagement (Brandstätter et al., 2013). Important variables that are involved in the disengagement process are emotions as incentives, as information, and as regulatory mechanism (Heckhausen et al., 2019) as well as detecting goal progress (Ghassemi et al., 2021) and being self-aware (Kreibich et al., 2022). While conscious mechanisms seem to play an important role in the goal disengagement process (e.g., reasons for disengaging, awareness of goal attainability and desirability), a conscious decision seems neither necessary to start the disengagement process, nor sufficient to end it (Rühs et al., 2022). However, we do not know whether the entire goal disengagement process could be unconscious until one realizes that one has already disengaged emotionally and behaviorally. In any case, one could assume that a conscious decision to disengage may foster the disengagement process (Holding et al., 2022). Moreover, the realization that one has already disengaged emotionally and behaviorally (compared to disengaging without acknowledging it) might play a role in how people allocate their resources in comparable future situations. In contrast, a conscious element might be a necessary but not sufficient condition to conclude the disengagement process. These considerations merit further investigation in future research.

Besides knowing some constructs involved in the goal disengagement process, prior research has also identified some moderating factors of this process: Self-control can foster or hinder goal disengagement, depending on boredom and self-awareness. Implementation intentions can help to disengage from a goal, particularly if one had previously defined specific criteria when to disengage. Goal motivation seems to play a more complex role: On the one hand, it appears to be more difficult to disengage from an autonomous or self-concordant goal (Ntoumanis et al., 2014). On the other hand, disengaging for autonomous *reasons* seems to promote progress in disengaging from a goal (Holding et al., 2022). Moreover, autonomous goal-motivation negatively (Holding et al., 2017) predicts action crises, while controlled goal-motivation positively predicts action crises (Holding et al., 2021), and they partially explain the link between mindfulness and action crises (Marion-Jetten et al., 2022). However, future research has yet to disentangle the manifold role that autonomous and controlled motivation seem to play in the different stages of the disengagement process. Another open question remains whether mindfulness and emotion regulation (Marion-Jetten et al., 2021, 2022) are only helpful in preventing action crises or whether they also help to disengage from any goal or only from certain goals.

We also know that the availability of alternative goals plays an important role and that people differ in this regard. For example, it is easier for people tending to avoid boredom and with escape tendencies to reengage in new goals. Moreover, implementation intentions foster reengagement. However, we know less about how disengagement and reengagement influence each other. On the one hand, reengaging in a new goal might make it easier to let go of an old goal. On the other hand, the feeling of closure from having disengaged from an old goal might be a crucial step for opening up to new goals. Whether disengagement helps reengagement or vice versa might also depend on the level one struggles to disengage. For example, if one cannot let go of a goal affectively, it might be helpful to reengage in new goals and enjoy their emotional benefits. This might make it then easier to affectively disengage from the old goal. In contrast, if one does not manage starting new goals, disengaging behaviorally from an old goal might help to reengage in new goals. These questions need to be further developed and investigated in future research.

Finally, we know that social relationships can constitute favorable or unfavorable conditions for goal disengagement. As such, romantic partners serve as role models for persisting (or not) in goal pursuit, but also in disengaging from futile tasks. Moreover, social support may have more negative consequences during an action crisis than otherwise, while, at the same time, not having fewer positive consequences either (Light & Chodos, 2022). Again, future research should aim at comparing the impact of social relationships for action crises with actual goal disengagement and examine social support explicitly directed at goal disengagement in contrast to supporting goal pursuit.

In sum, several factors identified as being relevant for goal engagement have also been identified as being relevant for goal disengagement. Maybe contrary to expectation, it is not their opposite expression that is related to successful goal disengagement. Their effect is rather dependent on other factors such as monitoring of goal progress, availability of alternatives, or the goal for which they are used. Future research could systematically investigate the conditions under which the same construct facilitates one or the other process.

In this special issue, we present studies using cross-sectional, prospective, and longitudinal studies as well as experimental studies. While correlational studies oftentimes focus on personal goals with a longer time perspective, laboratory studies usually induce goals with a shorter time perspective and less personal relevance. Although experimental designs are needed to draw causal conclusions, the question of comparability and generalizability of measured processes and identified influential factors arises. The field would benefit from combining experimental and (intensive) longitudinal designs (e.g., Neubauer et al., 2018) to carve out commonalities and differences.

In this vein, it would also be very fruitful to integrate research from cognitive psychology oftentimes based on experimental paradigms. For example, Hommel (2022) introduces an account of goals and their pursuit within the Theory of Event Coding (Hommel et al., 2001; Hommel, 2019) and its related representational assumptions. Based on this account, he posits that people develop different event files containing action-effect codes. These event files guide behavior and are activated by selection criteria associated with various sources (e.g., biological drives, acquired needs, instructed aims). These criteria vary in strength inter- and intra-individually. An event file is selected in a competitive process based on the best match (i.e., satisfaction of various criteria). Given that Hommel (2022) argues against goals as consisting of a coherent structure (and questions the necessity of the goal concept and Rubicon model in general), it raises the question in which sense goals need to or can be disengaged from and how disengagement could be conceptualized within the proposed account if the selected event file does not contribute to achieving the desired state.

Although Hommel (2022) does not explicitly consider goal disengagement in this account, his previous work on the Yin and Yang of action control (Hommel, 2015) provides some insight. Here, he argues that optimal action control consists in finding a balance between persistence and flexibility, that is, goal maintenance and openness to goal change (see also Cools & D’Esposito 2010; Dreisbach & Fröber, 2019). Within his Metacontrol State Model he proposes that several alternative actions compete for selection. A stronger leaning towards flexibility (and, thus, potential goal disengagement) is implemented by a lower top-down support for selecting the goal-relevant action alternative and/or a lower strength of mutual inhibition between alternatives. Hommel (2015) describes several factors including genetic predispositions, learning, and personal experience that shape the antagonistic processes in producing situational as well as dispositional differences in the emphasis on one or the other process. Integrating research strands predominantly presented in this special issue with research from cognitive psychology seems a worthwhile endeavor.

Moreover, while goal disengagement has received more research interest in the last 30 years, the focus was primarily on adulthood, in particular on individual differences (Baltes, 1997; Barlow et al., 2020; Brandtstädter & Greve, 1994; Heckhausen et al., 2019; Wrosch & Scheier, 2020). The few studies focusing on childhood and adolescence support the finding that goal disengagement processes dampen negative effects of unattainable goals on well-being and self-esteem (Greve & Enzmann, 2003; Marek et al., in press; Thomsen et al., 2015). Despite the focus on individual differences, the question of how goal disengagement capacities in general as well as individual differences in particular develop, has been neglected so far (Greve & Kappes, in press; Greve & Thomsen 2019; Heckhausen & Wrosch, 2016; Hommel, 2015; Kappes & Thomsen, 2022).

Overall, the question of when goal disengagement in contrast to goal engagement is warranted and adaptive remains to be given more thought. Given an individual differences perspective in adulthood, oftentimes criteria of well-being were used to test the functionality of goal disengagement. Assuming an objectively unattainable goal, disengaging from this goal certainly seems like a sensible response (but see *irrevocable goals*, Micheli & Castelfranchi, 2017). However, oftentimes the attainability of a goal and required resources are uncertain leaving the individual in the unknown about which way to proceed. While this state frequently arouses negative affect, while disengagement from such a goal decreases negative affect, enduring negative affect is sometimes functional to achieve a goal in the long run (i.e., adaptive effects of persistence). Therefore, disengaging might come with a prize, and disengaging frequently might have different effects in childhood than in old age. If goal disengagement is adaptive in the sense that it frees resources for future engagement with other goals, additional criteria of functionality could be examined in lieu of well-being indicators. Given that successful development is assumed to be the result of an interplay between engagement and disengagement processes (Baltes et al., 2006; Brandtstädter & Rothermund, 2002; Heckhausen et al., 2019), a criterion for the adaptiveness consists in the individual’s potential for future successful development in the form of being (better) able to cope with future challenges (Greve, 2015; Leipold & Greve, 2009). Obviously, this criterion is difficult to operationalize. However, it broadens the perspective to consider several outcome variables and their contextual dependence over time. In conclusion, properly disengaging from the right goals is about as challenging as choosing and then achieving the right goal in the first place.

## Footnotes

1 Brandstätter and Bernecker (2022) used “level” to refer to these different facets of the goal disengagement process. However, level implies some kind of hierarchy and can also be confused with a dual perspective of the mind-body problem, which we do not want to evoke here. Oettingen & Gollwitzer (2022) use the term “components”. This term refers to parts or elements of a larger whole and implies to a larger degree that all are actually necessary, mutually exclusive, and exhaustive to speak of disengagement. However, the point here is exactly that we do not know how affective, cognitive, and behavioral processes relate to each other. We merely want to argue for considering various facets in the goal disengagement process and to point out that it is conceptualized in different ways, providing findings that potentially lead to different conclusions. As we try to argue in the manuscript, to us, it is an open (conceptual) question how these facets relate to each other. We have therefore chosen the term “facet” to refer to something that might have various sides, where the number of facets is still undetermined and the (temporal) relationship between theses facets is still up to more research.

2 In order to allow recognizability, we retain the terms “frozen goals” and “shelved goals” as used in the respective manuscripts although they refer to closely related concepts. Davydenko et al., (2019) define frozen goals “as cognitive representations of desired end states that one maintains high commitment to achieving and that are achievable but are not actively pursued” (p. 2). Mayer & Freund (2022) use the term “shelving” to more strongly emphasize the *process* of (deliberate) temporary interruption of goal pursuit and its associated consequences. Whether using two terms for closely related concepts is actually warranted or whether one will prevail, needs to be seen.
